# Dataset on the literature on public participation and consensus building: Bibliography and meta-analysis of selected studies

**DOI:** 10.1016/j.dib.2023.109332

**Published:** 2023-06-22

**Authors:** Mahda Foroughi, Bruno de Anderade, Ana Pereira Roders

**Affiliations:** Department of Architectural engineering and technology, Faculty of Architecture and Built Environment, Technical University of Delft, the Netherlands

**Keywords:** Urban planning, Heritage planning, Multi-stakeholder decision making, Actor, Method, Cultural significance, Case study, Literature review

## Abstract

The data presented in this Data in Brief article offers an insight into the scientific literature on conceptual and empirical approaches to public participation and consensus-building. It consists of articles retrieved from the Scopus search engine which feature “public participation”, “consensus”, and “value and attribute” in the title, abstract, and author keywords. Information on the bibliography is recorded, namely title, author(s), year of publication, and source title. Metadata on how the articles were analyzed is provided in the dataset. From 121 publications, most literature (103) analyzes public participation through case studies. The studies were analyzed according to factors that were identified inductively and grouped in two categories: 1) public participation: actor, method, and level of public participation, and 2) consensus: approaches, conflict. The data is related to the research article entitled “Public participation and consensus-building in urban planning from the lens of heritage planning: A systematic literature review”. This paper focuses on the public participation factors as the factors on consensus are already explained in the main article. This paper shows which factors of participation were implemented in the analyzed studies. Given that, this article contributes to researchers and practitioners working on public participation because it reveals the diversity of approaches for consensus-building in public participation processes, which help them realize which level of participation they want to achieve and the means to reach it.

**Specification Table**

**Table:** 

Subject	Urban planning

Specific subject area	Consensus-building in heritage planning
Type of data	Table
How data were acquired	Systematic search in Scopus
Data format	Raw
	Analyzed
Description of data collection	First step: The potentially relevant studies were collected and pre-reviewed. This was done by searching various terms related to the research's three main concepts “public participation”, “consensus”, and “value and attribute”) in different combinations in the title, abstract, and author keywords in Scopus data base. Second step: a content-related selection of studies was conducted, leading to the selection of 121 studies for an in-depth analysis from 618 potentially relevant studies.
Data source location	Repository name: Mendeley
Data accessibility	Direct URL to data: https://data.mendeley.com/datasets/gxxzxyx7f6
Related research article	Foroughi, M., de Andrade, B., Roders, A.P. and Wang, T., 2023. Public participation and consensus-building in urban planning from the lens of heritage planning: A systematic literature review. Cities, 135, p.104235.

## Value of the Data

• Due to the systematic collection process and distinctive search terms, the dataset provides a unique detailed summary of the existing research on factors that affect consensus-building in a public participation process in urban planning, including heritage planning. The revealed factors are actors, methods, and levels of public participation.
• This dataset can be used to identify patterns and trends in the research, which can help researchers to develop hypotheses and research questions for their own studies. Besides scholars can replicate the studies to test the validity of the results or extend existing studies by considering the studies’ gaps (e.g, innovative distinctive methods, underrepresented actors, empowerment level of public participation).
• The data is analysed according to three theoretical frameworks which contribute to identifying the patterns in public participation case studies and makes the dataset more valuable for further analysis by scholars. Besides, this analysis illustrates the diversity of actors, methods, and levels of public participation in the analysed case studies.
• The dataset is useful in better illustrating the material used and data analysed in the related research article. Besides, the dataset contributes to future practice and research in the field.
• Researchers and practitioners that plan to investigate or design a participatory process can benefit from this dataset by (i) gaining an overview of relevant existing studies in the fields of urban planning and heritage studies that explore consensus-building in public participation process, (ii) gaining knowledge and comparing various practiced approaches to actors, methods, and levels of public participation, (iii) learning from shortcomings and possible limitations in the revealed factors.
• The dataset builds a structured baseline for further in-depth analyses of used participatory approaches and occurring shortcomings for studies on different geographical context.
• The dataset contains tables depicting the different implemented factors (e.g., actors, methods, and levels) to participation.

## 1 Objective

The original paper is a literature review paper which broadly analysed 121 publications on consensus-building and public participation. Although part of the data is delivered in the original paper, there are many data that are not directly provided. Therefore, this paper would promote open science and data accessibility of the original paper which would increase reproducibility by making data and the associated research more discoverable, opening doors for collaboration, and reducing duplication of effort.

## 2 Data Description

The dataset consists of four Excel spreadsheets that describe relevant studies in urban planning, with a focus on public participation, consensus-building, and cultural significance. The first spreadsheet contains metadata, including bibliographic details of all the articles such as the title, author(s), publication year, and journal, along with the geographical area(s) of focus. Additional metadata is provided in the following spreadsheets to identify the participation factors.

The second and third spreadsheets detail the first factor: actors involved in the case studies. The second spreadsheet provides raw data on group names mentioned in the studies, while the third spreadsheet shows the analyzed data over actors’ groups according to the actors’ framework (Pereira Roders, 2019). The fourth spreadsheet includes raw and analyzed data on the second and third factors: level of public participation, as specified by the IAP2 framework [1], and method of public participation.

The summary and interpretation of analysis is included in the research article entitled “Public participation and consensus-building in urban planning from the lens of heritage planning: A systematic literature review” [2].

## 3 Experimental Design, Materials and Methods

### 3.1 Scientific protocol for data collection

Conducting a comprehensive literature review was the primary objective of this study, which focused on three main concepts: “public participation,” “consensus,” and “values and attributes.” To locate relevant studies, a range of search terms was employed, as described in the main research [2]. Due to the limited number of publications addressing all three concepts, articles with at least two of the key terms in their title, abstract, or keywords were considered. Scopus, a peer-reviewed academic database, was used as the primary source of data in June 2019, with publications obtained from several fields, including Social Sciences, Engineering, Environmental Science, and Arts and Humanities. Initially, the abstracts of 618 articles were reviewed, and irrelevant studies were eliminated based on specific inclusion and exclusion criteria, such as content, language, and document type. Subsequently, 121 articles published between 1985 and 2019 were selected for analysis.

**Fig. 1:**
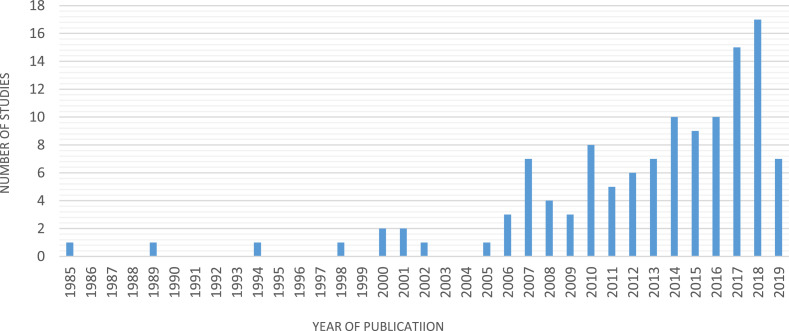
Annual distribution of all publication included in the database.

Fig. 1 presents the distribution of included publications in the database per year. Overall, the number of records has been increasing over the years. It should be noticed that low number of papers in 2019 could be because the data collection took place on June 2019 and only studies that are published before July 2019 were considered.

## 4 Scientific Protocol for Data Analysis

Thematic analysis was utilized in this study to identify the factors involved in public participation and consensus-building concerning cultural significance, following Nowell et al.'s procedure [3]. A data-driven inductive approach was used, and emerging themes were examined through initial coding and recoding, followed by a second set of codes. This analysis revealed the factors and sub-factors that contribute to public participation and consensus-building, which are categorized into two groups: 1) public participation - actor, method, and level of public participation, and 2) consensus - approach and conflict. This paper will focus on the factors of public participation.

To analyze the literature, three theoretical frameworks were employed that relate to the level of public participation, actors, and methods of public participation. The IAP2 (International Association for Public Participation) framework, which is based on the “ladder of public participation” [4], was used to rank the level of public participation between 1 to [1]: inform, consult, involve, collaborate, empower. This framework was chosen because it defines clear relationships, objectives, and techniques for each level of public participation, which helps in categorizing case studies. Additionally, the framework presented by Pereira Roders (2019) was used to classify actors, which identifies two main categories: public (politicians, policy makers, officers) and private (professionals/experts, daily users, occasional users), as it has been applied to several sample cities to analyze participatory heritage management practices.

Since there was no pre-existing framework for methods of public participation, this factor was classified based on a framework presented in the main paper [2]. As a result, the methods of public participation were divided into two main categories: data collection and data analysis. Qualitative, quantitative, and mixed methods were included within each category. While qualitative methods are more interactive, quantitative methods use mathematical methods and models to reach a consensus. Qualitative methods were classified as those that use digital tools, analog tools, or both digital and analog tools. All of the quantitative methods analyzed in this study used both digital and analog tools; hence, the quantitative methods do not have sub-categories.

## Ethics Statements

Ethics statement is not applicable in this paper as the work does not involve any use of human subjects.

## CRediT authorship contribution statement

**Mahda Foroughi:** Conceptualization, Methodology, Investigation, Visualization, Writing – original draft. **Bruno de Anderade:** Conceptualization, Methodology, Writing – review & editing. **Ana Pereira Roders:** Conceptualization, Methodology, Writing – review & editing.

## Declaration of Competing Interests

The authors declare that they have no known competing financial interests or personal relationships that could have appeared to influence the work reported in this paper.

## Data Availability

Dataset on the literature on public participation and consensus building: Bibliography and meta-analysis of selected studies (Reference data) (Mendeley Data)
